# A Novel Zebrafish *ret* Heterozygous Model of Hirschsprung Disease Identifies a Functional Role for *mapk10* as a Modifier of Enteric Nervous System Phenotype Severity

**DOI:** 10.1371/journal.pgen.1006439

**Published:** 2016-11-30

**Authors:** Tiffany A. Heanue, Werend Boesmans, Donald M. Bell, Koichi Kawakami, Pieter Vanden Berghe, Vassilis Pachnis

**Affiliations:** 1 Development and Homeostasis of the Nervous System Lab, The Francis Crick Institute, London, United Kingdom; 2 Laboratory for Enteric Neuroscience, TARGID, KU Leuven, Leuven, Belgium; 3 Crick Advanced Light Microscopy, The Francis Crick Institute, London, United Kingdom; 4 Division of Molecular and Developmental Biology, National Institute of Genetic and Department of Genetics, SOKENDAI (The Graduate University for Advanced Studies), Mishima, Shizuoka, Japan; Erasmus MC, NETHERLANDS

## Abstract

Hirschsprung disease (HSCR) is characterized by absence of enteric neurons from the distal colon and severe intestinal dysmotility. To understand the pathophysiology and genetics of HSCR we developed a unique zebrafish model that allows combined genetic, developmental and *in vivo* physiological studies. We show that *ret* mutant zebrafish exhibit cellular, physiological and genetic features of HSCR, including absence of intestinal neurons, reduced peristalsis, and varying phenotype expressivity in the heterozygous state. We perform live imaging experiments using a UAS-GAL4 binary genetic system to drive fluorescent protein expression in ENS progenitors. We demonstrate that ENS progenitors migrate at reduced speed in *ret* heterozygous embryos, without changes in proliferation or survival, establishing this as a principal pathogenic mechanism for distal aganglionosis. We show, using live imaging of actual intestinal movements, that intestinal motility is severely compromised in *ret* mutants, and partially impaired in *ret* heterozygous larvae, and establish a clear correlation between neuron position and organised intestinal motility. We exploited the partially penetrant *ret* heterozygous phenotype as a sensitised background to test the influence of a candidate modifier gene. We generated *mapk10* loss-of-function mutants, which show reduced numbers of enteric neurons. Significantly, we show that introduction of *mapk10* mutations into *ret* heterozygotes enhanced the ENS deficit, supporting *MAPK10* as a HSCR susceptibility locus. Our studies demonstrate that *ret* heterozygous zebrafish is a sensitized model, with many significant advantages over existing murine models, to explore the pathophysiology and complex genetics of HSCR.

## Author Summary

Hirschsprung Disease (HSCR) is a common congenital intestinal motility disorder diagnosed at birth by absence of enteric neurons in the distal gut, leading to intestinal obstruction that requires life-saving surgery. HSCR exhibits complex inheritance patterns and its genetic basis is not fully understood. Although well studied by human geneticists, and modelled using mouse, significant questions remain about the cellular and genetic causes of the disease and the relationship between neuron loss and defective intestinal motility. Here we use accessible, transparent zebrafish to address these outstanding questions. We establish that *ret* mutant zebrafish display key features of HSCR, including absence of intestinal neurons, reduced gut motility and varying phenotype expressivity. Using live imaging, possible in zebrafish but not in mouse, we demonstrate that decreased migration speed of enteric neuron progenitors colonising the gut is the principal defect leading to neuron deficits. By direct examination of gut motility in zebrafish larvae, we establish a clear correlation between neurons and motility patterns. Finally, we show that *mapk10* mutations worsen the enteric neuron deficit of *ret* mutants, indicating that mutations in *MAPK10* may increase susceptibility to HSCR. We show many benefits of modelling human genetic diseases in zebrafish and advance our understanding of HSCR.

## Introduction

The genetic basis of diseases exhibiting simple Mendelian inheritance can be readily uncovered, especially now using techniques available in the post-genomic era [1]. And, in the best examples, diseases such as cystic fibrosis and muscular dystrophy are well modeled using monogenic mouse mutants, enabling the diseases to be studied and new therapies to be tested [2, 3]. However, the genetic basis of human diseases exhibiting complex, multifactorial and/or polygenic inheritance is daunting to unravel. Additionally, conditions such as autism and schizophrenia, show spectra of phenotypes, making clear association between genotype and phenotype difficult [4]. Genetic studies of such diseases require advanced methodologies, including genome-wide association studies (GWAS), and this analysis throws up many possible candidate loci. And, perhaps expectedly, modeling these diseases has proven challenging (i.e. [5].

Hirschsprung Disease (HSCR), a common gut motility disorder (occurring in 1:5000 live births) characterized by defects in the enteric nervous system (ENS), is one such complex disease, being both multigenic and displaying varying expressivity [6, 7]. HSCR is diagnosed as an absence of enteric ganglia in varying extents of the distal colon, and affected newborns present with tonic contraction of aganglionic colonic segments, leading to accumulation of luminal contents and, if left untreated, toxic megacolon [8, 9]. Despite decades of genetic studies, twin challenges continue to drive research into studies of ENS development and the genetics of HSCR: firstly, understanding the developmental defects in the ENS that lead to intestinal aganglionosis that defines HSCR, and secondly, unraveling the complex genetics that control HSCR presentation.

The ENS is the part of the peripheral nervous system contained within the gut wall that controls intestinal motility, secretions and blood flow. Enteric neurons and glial cells are organized into interconnected ganglia situated between smooth muscle layers [10, 11]. The ENS derives mostly from vagal neural crest (NC) cells that invade the foregut during embryogenesis, hereafter called enteric NC-derived cells (ENCCs). Once within the gut wall, ENCCs proliferate extensively, migrate rostro-caudally and differentiate into vast numbers of neurons and glial cells organized into functional networks along the length of the gastrointestinal tract [12–14]. Overwhelming experimental evidence has demonstrated that the signaling pathway mediated by the RET receptor tyrosine kinase and the RET ligand GDNF, plays a critical role in ENS development, controlling survival, proliferation, migration and differentiation of ENCCs [12, 15–17]. Consistent with these findings, coding mutations of *RET* are detected in 50% of familial HSCR cases, while it has been suggested that non-coding regulatory mutations are likely present in most patients [8, 9]. Despite clear association between HSCR and *RET*, the cellular process(es) principally responsible for colonic aganglionosis is currently unknown. Furthermore, inheritance of HSCR is complex and not yet understood [8, 9]. For example, the non-coding mutations of *RET* associated with HSCR are common in the general population (~25%), indicating that they are not sufficient to lead to disease presentation [18]. Current models suggest that in most cases, individuals with non-coding *RET* mutations also carry modifier loci, many yet unknown, which contribute to HSCR presentation [18] and phenotype severity [6, 7]. Identification of such loci is a major challenge in HSCR research.

Transgenic mouse models have been critical in establishing key features of ENS development and HSCR disease [12, 13]. However several limitations are associated with these models, including a lack of simple heterozygous hypomorphic *Ret* mutations (as often observed in HSCR) phenocopying HSCR and it’s complex heritability, an inability to track ENS development in living embryos, an inability to examine *in vivo* intestinal motility in normal or disease states, and an absence of tractable models to screen for candidate HSCR susceptibility loci. To address these limitations, the genetically powerful and optically transparent free-swimming zebrafish larva has emerged as a viable alternative to mammalian models [19, 20]. Here we present an alternative model for HSCR disease in zebrafish and use it to address outstanding questions related to HSCR.

## Results

### Dose-dependent requirement for *ret* in ENS development

**Fig 1:**
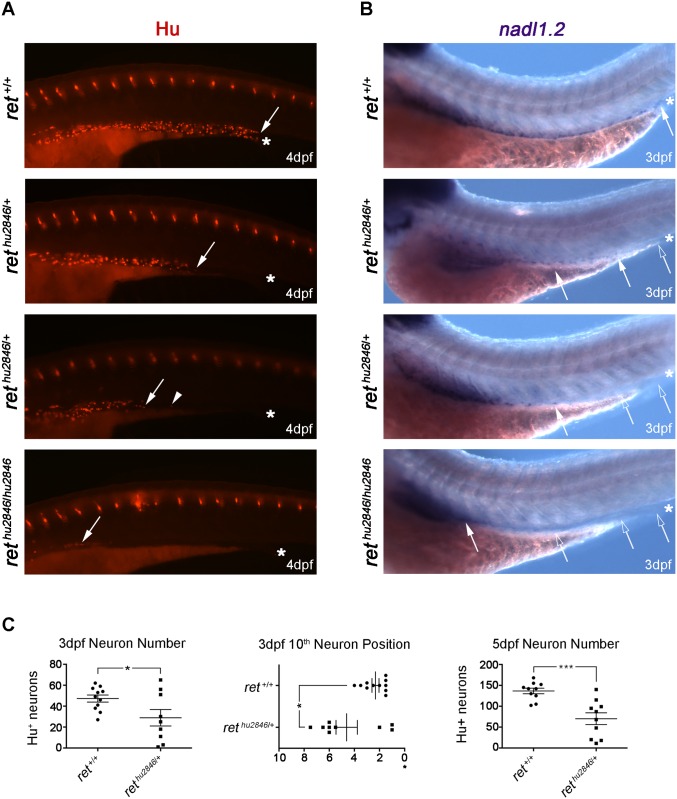
Loss of *ret* leads to dose-dependent reduction of ENS progenitors and neurons. (A-B) WT (*ret*^+/+^), *ret*^*hu2846*/+^, and *ret*^*hu2846/2846*^ larvae immunostained at 4dpf with HuC/D antibody to visualize enteric neurons (A) and processed at 3dpf by RNA *in situ* hybridization to detect *nadl1*.*2* expressing ENCCs (B). Asterisks indicate end of gut tube (anal pore), filled arrows and arrowheads denote position of last HuC/D^+^ neuron or *nadl1*.*2*^+^ ENCCs, and open arrows denote gut areas lacking *nadl1*.*2*^+^ ENCCs. (C) Number of HuC/D^+^ neurons in the distal gut was significantly reduced relative to WT counterparts, at 3dpf (WT: 47±3, *ret*^*hu2846*/+^: 29±8, p = 0.0281) and 5dpf (WT: 137±7, *ret*^*hu2846*/+^: 70±14, p = 0.0009). However, *ret*^*hu2846*/+^ larvae had a phenotypic range: some *ret*^*hu2846*/+^ larvae showed neuron numbers equivalent to WT, and other *ret*^*hu2846*/+^ larvae showed comparably fewer neurons than WT. Position of the 10^th^ most distal enteric neuron was also significantly altered in *ret*^*hu2846*/+^ larvae. Y-axis indicates somite lengths from end of the gut (*) (in *ret*^*hu2846*/+^ larvae 4.6±0.8 somite lengths from the end of the gut vs. 2.3±0.3 in WT, p = 0.012). Again, *ret*^*hu2846*/+^ larvae display a phenotypic range.

To establish a genetic model allowing us to explore Ret function throughout development, minimize experimental variation, and avoid potential off-target effects of Morpholino Oligos (MOs) [21], we used a TILLING strategy [22] to identify a zebrafish *ret* mutant allele. One allele, *ret*^*hu2846*^, contains a point mutation in the 5’ *ret* coding sequences, leading to a truncated, non-functional protein [23]. We first assessed the effect of *ret*^*hu2846*^ on gut colonization by enteric neurons. As expected, at 4dpf, HuC/D^+^ enteric neurons were detected in the intestinal bulb (a structure analogous to the stomach of terrestrial vertebrates) and along the full length of the intestine of WT (*ret*^+/+^) zebrafish larvae (Fig 1A) [19, 20]. In contrast, enteric neurons were almost absent from the gut of homozygous *ret*^*hu2846*/*hu2846*^ larvae, which contained only a few HuC/D^+^ neurons in the intestinal bulb (Fig 1A). These genetic studies are consistent with findings of earlier MO experiments [24, 25], and confirm requirement of *ret* for ENS development in teleosts.

Interestingly, in a cohort of *ret*^*hu2846*/+^ larvae, enteric neurons were restricted to rostral mid-intestine regions (Fig 1A). By counting HuC/D^+^ enteric neurons in the distal gut of 3dpf larvae and 5dpf larvae, we found that on average the number of enteric neurons was significantly reduced relative to WT counterparts (Fig 1C). However, this phenotype was incompletely penetrant, and other individual *ret*^*hu2846*/+^ larvae were less severely affected and, in some cases, were indistinguishable from WT (Fig 1C, S1 Fig). Indeed, several individual *ret*^*hu2846*/+^ larvae had more neurons than the average observed in WT larvae (Fig 1C). To determine whether neurons of the distal gut were uniquely affected, or whether observed deficits represented a more general effect on the ENS cell population, we compared the position of the 10^th^ most distal neuron between 3dpf *ret*^*hu2846*/+^ larvae and WT larvae. In a percentage of *ret*^*hu2846*/+^ larvae, the 10^th^ neuron position was equivalent to WT, but in the remainder it was positioned more anteriorly (Fig 1C), indicating a generalized, incompletely penetrant ENS phenotype. The observed gut colonization defects of *ret*^*hu2846*/+^ larvae contrast with the apparently normal colonization of the gut in heterozygous *Ret* mutant mice [26], and mirror colonic aganglionosis observed in patients with heterozygous *RET* mutations [27], and varying expressivity displayed by related individuals [6, 7]. This suggests that *ret*^*hu2846*/+^ larvae could provide an animal model for studying the pathogenesis and complex genetics of HSCR.

### ENCC migration deficits account for enteric neuron loss in *ret*^*hu2846*/+^ larvae

To determine whether reduction or absence of enteric neurons in distal gut regions of *ret*^*hu2846*/+^ and *ret*^*hu2846*/*hu2846*^ larvae, respectively, is caused by abnormal gut colonization by progenitors or their defective neuronal differentiation, we analyzed gut colonization by migratory neural crest-derived cells using the independent ENCC marker *nadl1*.*2* [24]. At 3dpf, *nadl1*.*2*-expressing cells were detected along the full length of the gut tube in all WT larvae analyzed (Fig 1B). In contrast, *nadl1*.*2*^+^ cells were found at varying positions in the gut of same stage *ret*^*hu2846*/+^ larvae and were absent from the gut of *ret*^*hu2846*/*hu2846*^ larvae (Fig 1B). Based on these experiments, we suggest that absence of differentiated neurons in the distal gut of *ret* mutant zebrafish reflects a failure of ENCCs to colonize the organ during development.

Defective gut colonization by ENCCs in *ret*^*hu2846*/+^ larvae may be accounted for by reduced progenitor pool size, associated with decreased cell proliferation or increased cell death, or compromised migratory potential of ENCCs, or a combination of these factors. To distinguish between these mechanisms, we took advantage of the unique potential provided by zebrafish to directly visualize migration of ENS progenitors *in vivo*. First, we explored the possibility that a binary reporter system used to label other neuroectodermal lineages could be used to label ENCCs. By crossing SAGFF234A transgenic fish that express a Gal4 transcriptional activator gene trap construct [28, 29] with UAS:GFP transgenic animals [28], we were able to label several neural lineages within the central nervous system and cells within the gut. Closer examination revealed GFP expression in ENCCs and virtually all enteric neurons (S2 Fig). Using this tool, we then examined whether reduced proliferation and/or increased cell death may contribute to compromised ability of ENCCs to colonize the gut. BrdU incorporation (to label cells in S-phase of the cell cycle) demonstrated that at 48hpf proliferation of ENCCs was indistinguishable between *ret*^*hu2846*/+^ and WT embryos (S3A–S3C Fig). Moreover no TUNEL^+^ cells were observed within the GFP expressing population of WT or *ret*^*hu2846*/+^ embryos (S3F–S3I Fig). These observations suggest that the colonization defect observed in *ret*^*hu2846*/+^ heterozygous larvae was unlikely to result from reduced size of the ENS progenitor pool.

**Fig 2:**
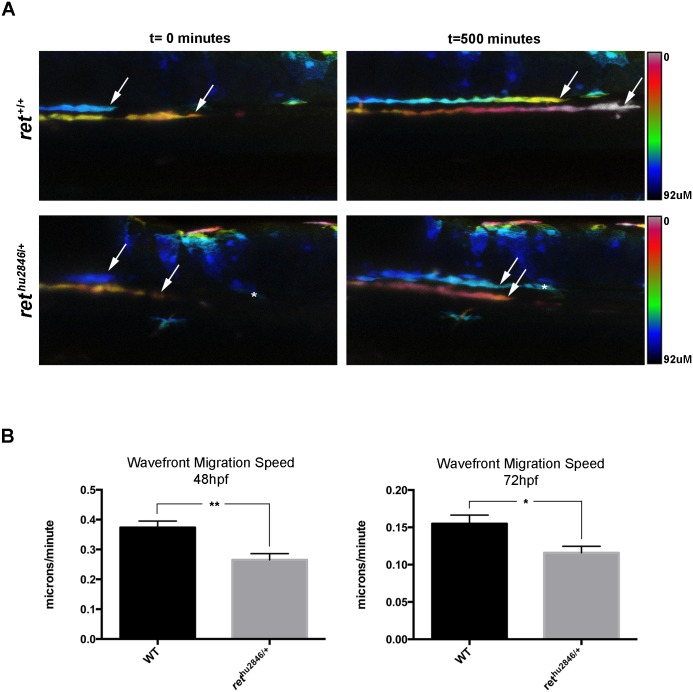
*ret*^*hu2846*/+^ larvae exhibit ENS progenitor migration deficits. ENS progenitor migration at the front of migration (wavefront) visualized in real time by virtue of the SAGFF234A;UAS:GFP background labeling ENCCs. (A) Representative still images from confocal time-lapse recordings (depth-coded to resolve 2 migratory streams) of WT (*ret*^+/+^) and *ret*^*hu2846*/+^ embryos at 0 minutes (48hpf) and 500 minutes (56hpf), show that distance travelled by the migration wavefront is decreased in *ret*^*hu2846*/+^ larvae relative to WT larvae. Asterisk denotes a GFP+ cell from outside the ENS linage that remains in the same position. (B) The calculated wavefront migration speed (microns/minute) is significantly reduced in *ret*^*hu2846*/+^ larvae relative to WT at 48–56hpf (p = 0.0022), and 72–80hpf (p = 0.0182).

In the course of these studies, we noticed that the shape of the group of advancing ENCCs was more rounded and less elongated in *ret*^*hu2846*/+^ embryos in comparison to WT embryos (S3D and S3E Fig), raising the possibility of a migratory deficit. We tested this idea directly by recording the migratory behavior of ENCCs using time-lapse confocal microscopy. We observed that at 48hfp, and 72hpf, the advancing speed of the front of the migratory population was reduced by 30% and 25%, respectively (Fig 2A and 2B, S1 and S2 Movies). We conclude that failure of enteric neurons to fully colonize *ret*^*hu2846*/+^ zebrafish guts likely results from decreased migratory speed of the ENCC population during gut colonization.

### Ret is required for normal intestinal motility

**Fig 3:**
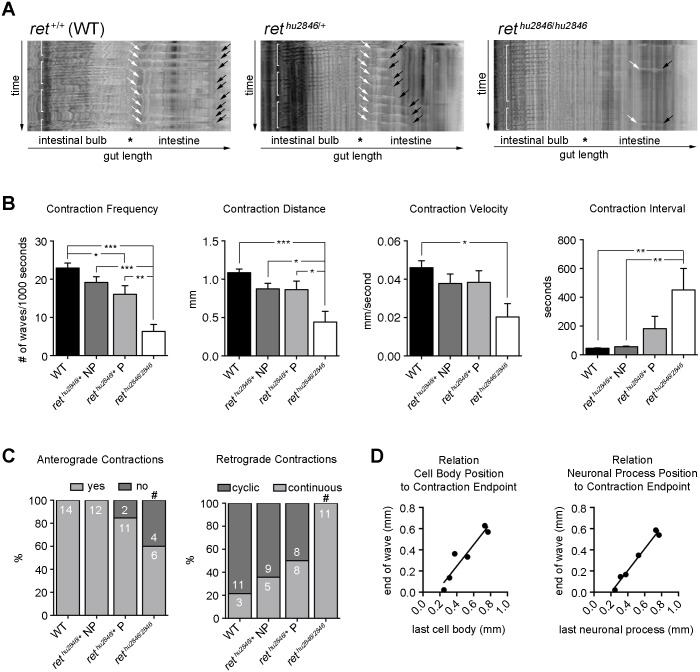
Intestinal motility is impaired in *ret*^*hu2846*/+^ and *ret*^*hu2846/2846*^ larvae. (A) Spatiotemporal maps (STMs) generated to quantify 400 second recordings of gut motility in live 7dpf zebrafish larvae. WT (*ret*^+/+^) larvae exhibit cyclical retrograde (anal to oral) motility patterns in the intestinal bulb (brackets), and anterograde (oral to anal) peristaltic waves in the intestine starting at the intestinal bulb/intestine junction (white arrows); black arrows denote motility end points. STMs of *ret*^*hu2846*/+^ larvae with ENS phenotypes and *ret*^*hu2846/2846*^ larvae show alterations in cyclical retrograde motility patterns (brackets) and frequency and distance of anterograde motility waves (white to black arrows). (B) Anterograde contraction characterization. Phenotypic (*ret*^*hu2846*/+^P) and non-phenotypic (*ret*^*hu2846*/+^NP) larvae identified immunohistochemically. Reduced peristaltic frequency in *ret*^*hu2846/2846*^ larvae and *ret*^*hu2846*/+^ P relative to WT (one-way ANOVA, p = 0.0004; Bonferroni’s post-hoc, p = 0.0001 and p = 0.0367), and also reduced in *ret*^*hu2846/2846*^ larvae as compared to *ret*^*hu2846*/+^ NP (p = 0.0002) and *ret*^*hu2846*/+^ P (p = 0.0035). Reduced contraction travel distance in *ret*^*hu2846/2846*^ larvae relative to WT (one-way ANOVA, p = 0.0005; Bonferroni’s post-hoc, p = 0.0002), and relative to both *ret*^*hu2846*/+^ P (p = 0.0232) and *ret*^*hu2846*/+^ NP (p = 0.0237). Reduced contraction velocity in *ret*^*hu2846/2846*^ relative to WT (one-way ANOVA, p = 0.0212; Bonferroni’s post-hoc, p = 0.0138). Increased contractions interval in *ret*^*hu2846/2846*^ larvae relative to WT and *ret*^*hu2846*/+^ NP (one-way ANOVA, p = 0.0078; Bonferroni’s post-hoc, p = 0.0053 and p = 0.0097). (C) Loss of anterograde contractions in *ret*^*hu2846/hu2846*^ larvae compared to WT and *ret*^*hu2846*/+^ NP (#, Fisher’s exact, p = 0.0198 and p = 0.028). *ret*^*hu2846/2846*^ larvae show retrograde motility changes relative to all other genotypes, with contractions losing cyclic patterns (Fisher’s exact, vs. WT: p = 0.0001, vs. *ret*^*hu2846*/+^ NP: p = 0.001, and vs. *ret*^*hu2846*/+^ P: p = 0.0082). (D) Equivalent analysis in the SAGFF234A;UAS:GFP background, expressing GFP in ENS cells and their processes, enables mapping of contraction endpoints relative to cell body position (GFP^+^HuC/D^+^ cells, r_s_ = 0.8857, p = 0.0333) and cell process (GFP^+^HuC/D^+^ process, r_s_ = 0.942, p = 0.0167). Black lines indicate linear fits.

To examine the effect of *ret* mutations on the motor output of the ENS, gut motility was video-recorded in 7dpf larvae. In agreement with previous observations [30], in the intestinal bulb of WT larvae, we observed churning retrograde (anal to oral) waves, while in the intestine we observed anterograde (propulsive oral to anal) peristaltic waves (Fig 3A, S3 Movie). Importantly, the reproducible character of recorded intestinal motility patterns, displaying regular directionality and frequency, consistent travel distances and fixed speed (Fig 3A), allows their quantification and detailed assessment of the effects of *ret* mutation. *ret*^*hu2846/hu2846*^ larvae showed severe defects in anterograde peristaltic activity in the intestine, including loss of contractions (Fig 3A and 3C, S5 Movie), reduction in frequency, distance, and velocity, and increase in interval (Fig 3B). Normal cyclical retrograde motility patterns were also lost in *ret*^*hu2846*/hu2846^ larvae and motility in the intestinal bulb became nearly continuous (Fig 3C). These findings clearly indicate that presence of enteric neurons throughout the intestine is required for normal gut motility.

Next we analysed effects of reduced *ret* expression on gut peristalsis in *ret*^*hu2846*/+^ larvae. Since the ENS gut colonization phenotype is variable in this genotype (S1B–S1D Fig), motility recordings were followed by immunostaining for HuC/D^+^ enteric neuron position. Spatiotemporal maps (STMs) of *ret*^*hu2846*/+^ larvae with full colonization (non-phenotypic, NP; S1B Fig) were largely indistinguishable from those of WT animals (Fig 3B and 3C). In contrast, STMs of *ret*^*hu2846*/+^ larvae with a clear colonization phenotype (phenotypic, P; S1C and S1D Fig) showed significant alterations in frequency of anterograde contractions (Fig 3B) and premature terminations of motility waves (Fig 3A, black arrows, S4 Movie). To determine more precisely the impact of neuronal loss on motility, we analysed *ret*^*hu2846*/+^ embryos in the SAGFF234A;UAS:GFP background, allowing us to map the endpoints of anterograde contractions relative to position of the last GFP^+^HuC/D^+^ cell body or neuronal process (see S2E Fig). We observed a tight correlation between peristaltic endpoints and position of HuC/D^+^ cells and their processes along the gut (Fig 3D), further indicating the requirement of enteric neurons for normal gut motility. These findings, together with our failure to observe deficits in neuronal differentiation or subtype composition in *ret*^*hu2846*/+^ larvae (S3K and S3M Fig), suggests that the altered motility patterns in *ret*^*hu2846*/+^ larvae result primarily from failure of colonization of the distal intestine by ENCCs and associated reduction of enteric neurons. Taken together these studies demonstrate that the *ret*^*hu2846*/+^ line provides an excellent model for analysing gut dysmotility associated with intestinal aganglionosis and HSCR.

### *mapk10* as a modifier gene for ENS development

**Fig 4:**
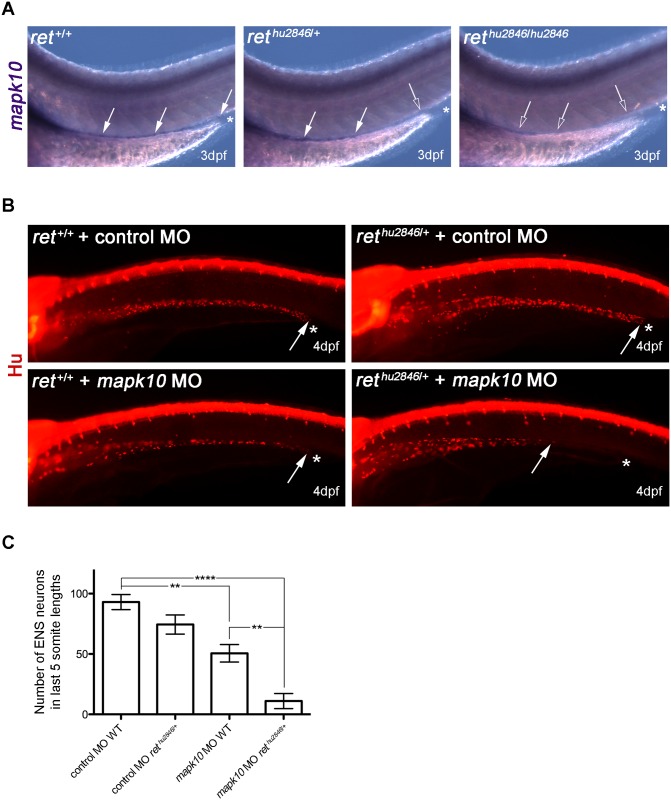
Using *ret*^*hu2846*/+^ as sensitized background to test role of *mapk10* as ENS development modifier gene. (A) RNA *in situ* hybridization shows *mapk10* expression correlates with location of the ENS in WT (*ret*^+/+^), *ret*^*hu2846*/+^, and *ret*^*hu2846*/hu2846^ 3dpf larvae (asterisks indicate end of gut, filled arrows indicate *mapk10*^+^ cells, and open arrows denote gut areas lacking *mapk10*^+^ cells). (B) MO gene knock-down of *mapk10* (using a splice blocking MO, *mapk10*MO) performed on embryos from WT x *ret*^*hu2846*/+^ cross to allow knock-down in both WT and *ret*^*hu2846*/+^ embryos, and compared to a control MO. Immunostaining with HuC/D at 4dpf shows that most severe phenotypes are observed when *mapk10*MO is injected into *ret*^*hu2846*/+^ embryos. Asterisks indicate end of gut tube (anal pore), and arrows denote position of last HuC/D^+^ neuron. (C) Quantification of neuron number in the last 5 somite lengths shows that injection of *mapk10*MO into WT embryos causes a modest, statistically significant reduction of enteric neurons in the gut relative to WT larvae injected with control MO (one-way ANOVA, p<0.001; Bonferroni’s post-hoc test, p = 0.0033), revealing a role for *mapk10* in normal ENS development. Injection of *mapk10*MO into *ret*^*hu2846*/+^ embryos causes severe reduction of enteric neurons, which is statistically different from either *ret*^*hu2846*/+^ injected with control MO (one-way ANOVA, p<0.001, Bonferroni’s post-hoc test, *P*<0.0001) or *mapk10*MO injected into WT embryos (one-way ANOVA p<0.001, Bonferroni’s post-hoc test p = 0.006).

Although changes in RET signaling underlie almost all cases of HSCR, the vast majority of genetic changes associated with this locus represent non-coding, regulatory mutations [18]. Although such mutations are thought to increase susceptibility to HSCR, full expression of the disease phenotype requires interactions with mutations in unlinked modifier genes [18]. To test the idea that the *ret*^*hu2846*/+^ genotype represents a sensitized genetic background suitable for phenotype-based screens for modifier loci affecting severity of ENS colonization phenotypes and manifestation of HSCR disease, we examined the effect of the *mapk10* gene on the ENS phenotypes in *ret*^*hu2846*/+^ larvae. Our focus on *mapk10* was directed for several reasons. First, *mapk10* was highlighted in a screen to identify novel vertebrate ENS markers and is highly expressed in the mammalian ENS [31]. Second, members of the Mapk family (also known as Jnks) act downstream of Ret in cancer cell signaling [32]. Third, *Mapk* signaling is impaired in RET^S697A^ mutant mice and Mapk inhibitors block ENCC migration in cultured guts [33]. Finally, copy number variants in *MAPK10* were identified in patients with HSCR [34]. By comparing the distribution of *mapk10* transcripts in the gut of WT, *ret*^*hu2846*/+^ and *ret*^*hu2846*/hu2846^ larvae, we first established that similar to mammalian embryos [31], *mapk10* is expressed in the zebrafish ENS (Fig 4A, see also S4 Fig). Next we performed gene knock-down experiments using a MO designed to block splicing of the *mapk10* exon4-intron 4/5 boundary (*mapk10*MO) and generate a truncated protein lacking all known conserved functional domains (S5A Fig). *mapk10*MO injections were performed on embryos resulting from crosses between WT and *ret*^*hu2846*/+^ animals, which was expected to generate WT and *ret*^*hu2846*/+^ embryos in a 1:1 ratio. Relative to control MO, injection of *mapk10*MO into WT embryos led to a statistically significant reduction of enteric neurons in the gut of 4dpf larvae (Fig 4B and 4C), suggesting a role for *mapk10* during ENS development. Interestingly, injection of *mapk10*MO into *ret*^*hu2846*/+^ embryos resulted in dramatic reduction of enteric neurons relative to *ret*^*hu2846*/+^ embryos injected with control MO or WT embryos injected with *mapk10*MO (Fig 4B and 4C). These results identify a role for *mapk10* during ENS development and suggest a genetic interaction between *mapk10* and *ret*, thus providing supporting for a model in which mutations in independently segregating loci modify *RET* activity and HSCR disease presentation.

### A novel *mapk10* mutant zebrafish exhibits mild ENS colonization phenotypes

While MOs are invaluable screening tools, their use can lead to non-specific effects, confounding experimental interpretations [21]. For this reason, we employed genome-editing techniques [35] to generate a *mapk10* mutant line and refine our analysis. Using TALEN [36], we generated, among other mutations, a 10 base pair deletion in exon 4 (*mapk10*^Δ10^) causing a frameshift in the open reading frame, leading to a premature stop codon and protein truncation, which lacks the entire protein kinase catalytic domain and all Mapk/Jnk conserved domains (S5B Fig). *mapk10*^Δ10/+^ and *mapk10*^Δ10/Δ10^ zebrafish were viable and fertile, without obvious morphological or behavioral phenotypes.

**Fig 5:**
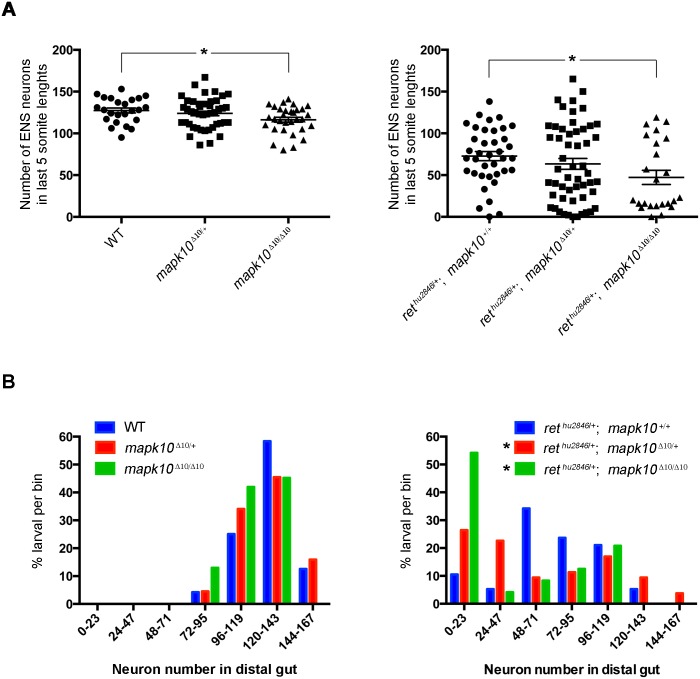
*mapk10* as candidate to account for varying expressivity in HSCR. (A) Enteric neuron number in the distal gut quantified in 4dpf larvae resulting from crosses between *mapk10*^Δ10/+^ and *ret*^*hu2846*/+^;*mapk10*^Δ10/+^ zebrafish. No difference was detected between WT and heterozygous *mapk10*^Δ10^ larvae, but *mapk10*^Δ10/Δ10^ larvae have a small, but statistically significant reduction in ENS cell number in the gut relative to WT (one-way ANOVA, p = 0.0442, Tukey post-hoc), indicating a role for *mapk10* in normal ENS development. No significant difference was detected between *ret*^*hu2846*/+^ and *mapk10*^Δ10/+^;*ret*^*hu2846*/+^ larvae, however, loss of *mapk10* in the *ret*^*hu2846*/+^ background leads to statistically significant reduction in neuron number (Welch’s one-way ANOVA, *p* = 0.048). (B) To examine phenotype distribution, individual zebrafish were binned according to neuron number in the distal gut. WT, *mapk10*^Δ10/+^ and *mapk10*^Δ10/Δ10^ larvae show normal distribution of phenotypes (blue, red, and green bars, respectively, Shapiro-Wilk normality test, for WT: p = 0.776, for *mapk10*^Δ10/+^: p = 0.910, and for *mapk10*^Δ10/Δ10^: p = 0.1149). Although *ret*^*hu2846*/+^ larvae guts are phenotypic, they display a range of neuron numbers in the distal gut region, reflecting normal distribution of mild and progressively more severe colonization phenotypes (Shapiro-Wilk normality test, p = 0.5720, blue bars), counts of neuron number in *ret*^*hu2846*/+^;*mapk10*^Δ10/+^ (red bars) and *ret*^*hu2846*/+^;*mapk10*^Δ10/Δ10^ larval guts (green bars) exhibit a non-normal distribution pattern (Shapiro-Wilk normality test, for *ret*^*hu2846*/+^;*mapk10*^Δ10/+^: p = 0.0054, and for *ret*^*hu2846*/+^;*mapk10*^Δ10/Δ10^ p = 0.0014), and counts of enteric neuron number in *ret*^*hu2846*/+^;*mapk10*^Δ10/Δ10^ larvae, showed a statistically significant increase in standard deviation compared with *ret*^*hu2846*/+^ larvae (Brown-Forsythe, 0.0445).

To further examine the role of *mapk10* in ENS development, we compared enteric neuron number in the distal gut of 4dpf WT, *mapk10*^Δ10/+^ and *mapk10*^Δ10/Δ10^ larvae. Although no difference was detected between WT and heterozygous *mapk10*^Δ10^ larvae, *mapk10*^Δ10/Δ10^ larvae showed a relatively small, but statistically significant reduction in ENS cell number in the gut relative to WT (Fig 5A). In all genotypes, the percentage of animals containing a given range of enteric neurons in the distal gut follows a normal distribution (Fig 5B). Together, these observations are consistent with observed effects of *mapk10*MO injections and confirm a role for *mapk10* in normal ENS development. While both *mapk10* loss of function experiments show the same effect to reduce ENS neuron number, the *mapk10* MO has a more profound effect on ENS colonization than is observed in the homozygous *mapk10*^Δ10^ (compare Figs 4C to 5A). One possible explanation is that non-specific defects caused by MOs, such as cell death and developmental delays, may exacerbate the phenotype [21]. Alternatively, it has recently been suggested that compensatory mechanisms may occur in zebrafish genetic mutants, leading to milder phenotypes in genetic mutants than those generated by MOs [37]. Thus, these observations underscore the utility in employing a range of functional assays in order to validate results.

### Genetic analysis confirms *mapk10* as modifier gene for ENS development

To test for genetic interactions between *ret* and *mapk10*, we next examined combined effects of *mapk10*^Δ10^ and *ret*^*hu2846*^ mutations on enteric neuron numbers. No significant difference was detected between *ret*^*hu2846*/+^ and *mapk10*^Δ10/+^;*ret*^*hu2846*/+^ larvae (Fig 5A). In contrast, significant differences were observed in mean neuron number between *mapk10*^Δ10/Δ10^;*ret*^*hu2846*/+^ and *ret*^*hu2846*/+^ larvae (73 neurons in *ret*^*hu2846*/+^ vs. 47 neurons in *mapk10*^Δ10/Δ10^;*ret*^*hu2846*/+^) (Fig 5A). These observations are consistent with effects of *mapk10*MO and further indicate a role for *mapk10* in ENS development.

Interestingly, while the recorded number of neurons in the distal gut of *ret*^*hu2846*/+^ larvae showed a normal distribution pattern, the phenotypes of *ret*^*hu2846*/+^;*mapk10*^Δ10/+^ and *ret*^*hu2846*/+^;*mapk10*^Δ10/Δ10^ larvae exhibited a non-normal distribution pattern (Fig 5A and 5B). Moreover, counts of enteric neurons in *ret*^*hu2846*/+^;*mapk10*^Δ10/Δ10^ larvae, showed a statistically significant increase in standard deviation compared with *ret*^*hu2846*/+^ larvae (Fig 5A and 5B), with a substantial cluster of severely affected individuals. Specifically, 54% of *ret*^*hu2846*/+^;*mapk10*^Δ10/Δ10^ larvae had severe phenotypes (less than 24 neurons), relative to only 10% observed amongst *ret*^*hu2846*/+^ larvae (Fig 5A and 5B). Taken together these results suggest that loss of *mapk10* in *ret* heterozygous larvae can lead to increased severity of *ret*-dependent ENS phenotypes, thus highlighting *MAPK10* as a candidate gene contributing to expressivity of aganglionosis and manifestation of HSCR.

## Discussion

We demonstrate here that Ret signaling is essential for gut innervation of teleosts and employ zebrafish as a model system to gain mechanistic insight into ENS development and the pathogenesis of congenital intestinal neural deficits.

Specifically, we show that the *ret*^*hu2846*^ mutant zebrafish line lacks enteric neurons in distal gut segments (the defining feature of HSCR), that individual larvae show varying expressivity of this cellular defect (a well-established feature in the complex genetics of HSCR), and that absence of enteric neurons correlates with defective gut motility (equivalent to the dysmotility associated with the megacolon of HSCR patients). By combining the *ret*^*hu2846*^ line with novel transgenic tools and *in vivo* imaging, we provide evidence that reduced migration of ENCCs is a major contributory factor for distal intestinal aganglionosis. Finally, we demonstrate that this HSCR model can be used as a sensitized background to identify genes that influence ENS phenotype expressivity and therefore are candidate HSCR susceptibility loci. Our current studies focus on *mapk10*, demonstrating its requirement for development of the full complement of enteric neurons and we show that mutations in this gene can enhance the loss of ENS phenotype of *ret*^*hu2846*^ heterozygous larvae.

### Decreased ENCC migration speed as major contributor to HSCR-like phenotypes

Assembly of functional intestinal neural circuits depends on overlapping developmental processes, namely survival and proliferation of ENCCs, migration along the gut, and differentiation into multiple neuronal and glial cell types [12, 13]. Although deficits in any of these processes could contribute to intestinal aganglionosis [12, 13], their relative contributions to ENS development remain unclear. By combining the power of zebrafish genetics with the optical accessibility of zebrafish, we demonstrate here that reduced migration speed of ENCC streams is a major determinant of ENS loss in the distal gut of *ret*^*hu2846*/+^ larvae. These experiments corroborate recent studies using murine organotypic cultures challenged with pharmacological inhibitors of *Ednrb* signaling, which concluded that decreased ENCC migratory speed is a principal mechanism of aganglionosis in this paradigm [38]. Since mutations in *RET* and *EDNRB* are the most frequent genetic changes encountered in familial cases of HSCR (EDNRB mutations detected in ~5% of HSCR patients) [8], our data, together with those of Young et al. [38], suggest that reduced ENCC migration is a key pathogenic mechanism underlying failure of ganglia formation in the distal colon of HSCR patients. Although we obtained no evidence of cell proliferation defects or apoptotic cell death in *ret*^*hu2846*/+^ embryos, potential contributions of these processes in HSCR-like phenotypes remain to be determined. Nevertheless, our current studies highlight differential sensitivity of distinct cellular processes to reduced levels of Ret activity.

### Organized motility patterns linked to presence and position of enteric neurons

Although the importance of the ENS in regulating intestinal motility is well established [11], the gut dysmotility observed in mouse models of aganglionosis remains poorly characterized *in vivo*. By conducting systematic analysis of video recordings of live zebrafish larvae, we demonstrate here that *ret*^*hu2846*^ homozygous larvae show dramatic deficits in intestinal motility, highlighted by the almost complete elimination of anterograde peristaltic waves. This finding echoes the loss of intestinal motility observed in *sox10*^*colourless*^ homozygous zebrafish lacking enteric neurons [39] and motility changes observed in WT larvae treated with the sodium channel blocker tetrodotoxin (TTX) [30]. Milder defects were observed in *ret^hu2846^* heterozygous larvae, and likely mirror the dysmotility observed in HSCR patients [40]. The tractable number of enteric neurons, and the ability to clearly visualize neuronal somata and their projections along the gut enabled us to use individual *ret*^*hu2846*^ heterozygous larvae representing different degrees of distal aganglionosis to map with high resolution the endpoints of motility waves relative to the position of the last neuron and/or neural projections. The close association we show between organized peristaltic motility and neuron position builds on previous correlations observed between ganglionic states and motility [41] and makes clear the requirement of local control of muscle by neural networks. The relationship between ganglionated state and capacity to generate motility waves is of clinical interest [42]. Our studies may explain why impaired motility (including constipation) is observed in HSCR patients after transitional zone pull-through (TZPT) (where part of the hypoganglionated gut remains after surgery) [43]. Additionally, our observations argue that the promise of successful treatment of long-segment HSCR by stem cell transplantation [44–47], would require colonization of aganglionic gut segments along their full length. Further work using such zebrafish models has the potential to establish links between states of ganglionosis and their capacity to mediate peristalsis and other ENS regulated processes, and determine, for example, the minimal neurofunctional unit required to restore organized motility. Finally, we suggest that STM analysis of actual intestinal motility can form the basis of highly sensitive assays to assess the role(s) of genetic pathways and environmental (including luminal) factors (i.e. [48] on the assembly of neuronal circuits controlling the motor behavior of the intestine.

### *ret*^*hu2846*^ heterozygotes as powerful tool in identifying HSCR susceptibility genes

*ret*^*hu2846*/+^ larvae reproduce the aganglionosis phenotype of HSCR patients with *RET* mutations who are invariably heterozygous at this locus [6, 7, 18], and contrasts *Ret*^+/-^ mice, which show normal development of the ENS [26]. The difference in length and surface area between mouse and human bowel has been cited as a possible reason for phenotypic discrepancy between the two species [7]. However, given the small size of the zebrafish larval gut, our experiments would argue instead that ENS lineages of the mouse are less sensitive to Ret activity levels relative to either human or zebrafish. A potential explanation for this difference is that mouse HSCR-like lines, including those with *Ret* mutations, are generally maintained on inbred (isogenic) backgrounds. In contrast, mutant zebrafish lines are maintained as outbred populations (due to effects of inbreeding depression) [49, 50], thus sharing with human populations the high degree of genetic heterogeneity associated with outbreeding. Consequently, zebrafish populations are expected to carry greater numbers of individual mutations, with limited genetic pressure to eliminate mutations that would (perhaps even minimally) affect Ret levels. It has been proposed that a full HSCR phenotype results only when >50% of RET function is lost, whether caused by structural or regulatory mutations in *RET* (as is the case of numerous known coding mutations [8] or the common *RET* enhancer mutation *rs2435357* [51], respectively, or from interactions with other susceptibility genes [52]). Since all *ret*^*hu2846*/+^ individual larvae express the same *ret* loss of function allele, observed variations in expressivity are either due to structural or regulatory mutations at the other *ret* allele, or mutations at other loci. Therefore, heterozygous *ret*^*hu2846*^zebrafish can be used to test the genetic basis of phenotype expressivity.

Human genetic studies and patient genome- or exome- sequencing have identified, in addition to *RET*, 13 HSCR-associated genes accounting for disease susceptibility [7, 16, 53, 54]. Yet, mutations in these genes account for only ~50% of familial HSCR cases and 20% of sporadic HSCR [51], indicating that novel loci responsible for the missing heritability remain to be uncovered. Here we have used the *ret*^*hu2846*/+^ background to provide further evidence that *MAPK10* is a HSCR susceptibility locus [34]. By generating a novel *mapk10* deletion in zebrafish, we demonstrate that *mapk10* is required for normal ENS development in vertebrates and *mapk10* mutations dramatically increase severity of ENS phenotypes observed in *ret*^*hu2846*^ heterozygous larvae. We hypothesize that in animals in which the influence of *mapk10* mutation on phenotypic expressivity is strong, loss of Mapk10 activity may affect, directly or indirectly, Ret signaling. In contrast, in animals in which expressivity is not significantly altered by loss of *mapk10*, additional factors associated with genetic variation may buffer potential effects of *mapk10* deletion. Recent bioinformatics analysis of mouse gene expression profiles in the ENS hypothesize a role for Mapk10 downstream of RET in HSCR [55]. Further study of these interactions, for example in experiments testing the ability of putative downstream factors to rescue of the *ret* mutant phenotype, can build our understanding of gene regulatory and signaling modules influencing ENS development and impacting on HSCR.

Determining the mechanism by which interacting factors lead to phenotype expressivity will be of significant interest. In the case of *mapk10*, the exacerbation of *ret* mutant phenotypes may be caused by causing further delays to progenitor cell migration, by inducing cell death, or reducing ENCC proliferation. It is also possible that a combination of these negative influences leads to the observed phenotypes, since proliferation is itself a force driving ENCC migration [56, 57]. Known functional associations of Jnk/Mapk signaling include roles in differentiation, apoptosis, and migration. It is believed that the diverse roles are due to the large number of Jnk interacting factors [58]. The suggestion from work in mouse is that migration defects is the major consequence of loss of Jnk signaling, since RET^S697A^ mutant mice, which have impaired Mapk signaling (among other signaling defects), exhibit impaired cell migration without alterations in cell proliferation or survival [33]. However, future study of the *mapk10* mutant zebrafish will enable a direct examination of the role(s) of Mapk10 in ENS development and in ENS phenotype expressivity.

Because of the multifactorial etiology of HSCR, disease risk cannot be predicted, even in cases of familial HSCR [51, 59]. For this reason genetic counseling is not usually conducted for HSCR, however, surgical strategies are generalized. In the age of personalized medicine, it would be reasonable to expect that if patients could be catalogued based on genetic profiles, then treatment plans, be they surgical or using cell replacement therapies, could be assessed for efficacy amongst different classes of patients. Then, future treatment strategies could be tailored to cope with specific needs of individual patients. To attempt such an approach, the impact of gene mutations on disease expressivity must be understood. Further studies in zebrafish will play a significant role in identifying key regulators of ENS development and their roles in HSCR phenotype expressivity.

## Materials and Methods

### Animals

All animal experiments were carried out in compliance with the Animals (Scientific Procedures) Act 1986 (UK) and in accordance with the regulatory standards of the UK Home Office (Project Licence 70/7644 to VP). Experiments were also approved by the Animal Welfare and Ethical Review Body (AWERB) of the MRC National Institute for Medical Research/The Francis Crick Institute. Maintenance of zebrafish stocks was performed as described [60]. Embryos and larvae were maintained at 28°C in an illuminated incubator on a 14 h:10 h light:dark cycle. Stages of embryos and larvae were assigned in hours or days post fertilization (hfp or dpf, respectively) according to morphological features [61]. Larvae from 5 to 7dpf were fed ZM-000 (ZM Systems) daily within 30 minutes of onset of light cycle. Tubingen LongFin (TL) was used as background strain for all experiments. In specific experiments (described below), embryos were reared in 0.15M 1-*phenyl 2-thiourea* (PTU) from 24hpf to inhibit melanization. The *ret*^*hu2846/*+^ line and its genotyping has been described previously [23]. The SAGFF234A transgenic fish was identified by a large scale genetic screen using a gene trap construct containing Gal4FF [28, 29], and in the case of SAGFF234A, the Gal4 expressing gene trap construct was integrated within the *chrm2a* gene on Chromosome 4 encoding the cholinergic receptor, muscarinic 2a. The UAS:GFP transgenic fish was described previously [28]. The *mapk10*Δ*10* line was genotyped as follows: PCR product amplified with primers fwd- ATCTCCACACAGGGTTTTGG and rev- CTGAATGTGAATACTCACCACACT, digested with *KpnI*, and analyzed by gel electrophoresis to detect *KpnI* site presence (WT allele, 135 and 65bp bands) or absence (mutant allele, 200bp band).

### RNA *in situ* hybridization and antibody staining

*RNA in situ* hybridization was performed as described previously using a *nadl1*.*2* probe [24] on PTU treated embryos. Probe for *mapk10* was generated by RT-PCR amplification using orthologue-specific [62]primers against NM_001037701.1 (GATTCAGCATCGCAAACTCA and GCCGAAATCCAAAATCTTCA amplify an 837bp fragment corresponding to 5’UTR and 5’cds of *mapk10*). The product was cloned into pGEMT-Easy and sequence verified, named *mapk10*-3, linearized with *SalI*, and transcribed with T7 Polymerase. Immunostaining on embryos and larvae was performed essentially as described [63], except that following overnight fixation in 4% PFA at 4°C, samples were washed 3 times in PBS containing 0.1% TritonX (PBT), equilibrated in water for 1 minute, permeabilized in acetone at -20°C, re-equilibrated in water for 1 minute, and washed in PBT before blocking, incubation with primary antibodies, washing and incubation with secondary antibodies as described [63]. Immunostaining after RNA *in situ* hybridization was performed after post-fixing processed samples for 20 minutes, washing 5 times in PBT, and then incubating with primary antibodies, with all subsequent steps performed as above. Antibodies used as follows: Hu (mouse, Life Technologies A21272, 1:200), GFP (chick, Abcam ab13970, 1:500), BrdU (rat, Abcam, ab6326, 1:200), 5-HT (rabbit, Sigma, 1:2000) and appropriate Alexa Fluor conjugated secondary antibodies (Molecular Probes).

### BrdU incorporation for cell proliferation analysis

Embryos were incubated with BrdU for 30 minutes, essentially as described [64] and processed for immunostaining, as above, except embryos were reared in PTU, and following fixation, dehydrated in methanol, rehydrated, washed in PBT, digested in 10mg/ml proteinase K for 10 minutes, washed in PBT, and post-fixed in 4%PFA for 20 minutes. Embryos were then washed in water, equilibrated in 2N HCl, before denaturing for 1 hour in 2N HCl at room temperature, neutralizing with two 10 minute 0.1M sodium tetraborate washes, and washing in PBT before blocking and immunostaining as above.

### TUNEL staining for apoptotic cell death analysis

Apoptotic DNA fragmentation detected using the TUNEL method. Performed after immunostaining, as above, essentially as described [65] and using the ApopTag Red *In Situ* Apoptosis Detection Kit for direct detection of DNA fragmentation.

### Time lapse imaging of ENS progenitor cell migration

Embryos were reared in PTU until 48hfp, lightly anaesthetized (0.15mg/mL Tricaine in fish water), and mounted in embryo arrays [66] embedded in 0.6% low melt temperature agarose (made in 0.5x Danieau’s solution with PTU and Tricaine). Time lapse imaging was performed for 36 hours on a Leica TCS SP5 confocal, with z-stacks collected for each embryo at maximal acquisition speed for the imaging set up (frame interval 374 seconds). Fiji [67, 68] with Trackmate plugin [69] was used to calculate travel distance and formulate migration speed of the migratory wavefront (defined as the position of the most distal GFP+ cell).

### ENS neuron counting

Quantification of the number or position of neurons in the distal gut was standardized using tissue landmarks to define gut lengths. In most assays, HuC/D^+^ ENS neurons were counted, starting from the anal pore and continuing towards the intestinal bulb until 5 or 10 somite lengths had been covered, for 4dpf or 3dpf larvae, respectively. Additionally, the position of the 10^th^ most distal HuC/D^+^ ENS neuron in 3dpf larvae was scored relative to somite lengths from the anal pore.

### Video imaging and analysis of intestinal motility

Embryos were reared without PTU. Following feeding at 5dpf and 6dpf, larvae were transferred to a fresh dish on the evening of the 6^th^ dpf, to enable gut motility to be visualized at 7dpf without luminal contents. Individual larvae were transferred into fish water with anesthetic (0.15mg/mL Tricaine in fish water) and laid, free-floating in lateral view in an agarose trough, and imaged at 2.5 frames per second for 16 minutes with a QICAM-Fast camera using QCapture Pro 6.0 software (Q-Imaging). Analysis of the movies was performed using Igor Pro (WaveMetrics) with custom-written algorithms, allowing movement of the gut wall to be computed and mapped over the imaging period. Spatiotemporal maps were generated and used to determine the presence and direction of contractions and contraction frequency (waves 1000 s^-1^, travel distance (mm), velocity (average speed over total propagated distance; mm s^-1^), and interval (s), similar to previous methods [30].

### Morpholino oligo injection

All *mapk10* sequence information based on *Ensembl* Zv9 build. The *mapk10* splice-blocking morpholino oligo (MO) (GeneTools LLC) *mapk10* MO targets the intron3/4-exon4 splice junction (TGCCAAAACCCTGTGTGGAGATAAA), leading to excision of exon 4, confirmed by sequencing. Control embryos were injected with Gene Tools standard control MO (CCTCTTACCTCAGTTACAATTTATA). Efficacy of *mapk10* MO was tested by RT-PCR essentially as described [24] with primers from exons 3 and 7 (TGTGAAGATTGCCTTTTGTCA and AATGCAGGTGTTTGATTCCA) to amplify a 500 bp band or a 330bp band if exon 4 excision has occurred. Bands of expected sizes were gel isolated and sequence verified as described [24].

### TALEN gene targeting

TALEN design and assembly was performed using Golden Gate TALEN assembly, according to protocols from TAL Effector Nucleotide Targeter 2.0 (Cornell University, http://boglab.plp.iastate.edu) [70, 71] as follows: TALEN target site T CCACCTTCACGGTTCTCAAA cggtaccaaaatctaaa GCCCATTGGTTCTGGGGCTC A (including a Kpn1 site), *mapk10* TAL left HD HD NI HD HD NG NG HD NI HD NN NN NG NG HD NG HD NI NI NI, *mapk10* TAL right, NN NI NN HD HD HD HD NI NN NI NI HD HD NI NI NG NN NN NN HD. Final plasmids used, pCS2-TALDD and pCS2-TALRR, as described [36]. HRM screening protocols essentially as described [36], using primers fwd: ATTCCACCTTCACGGTTCTC, rev: CTGAATGTGAATACTCACCACACT. High fidelity PCR amplification, cloning and sequencing of the *mapk10* target site of *mapk10*Δ*10* founder zebrafish, confirmed a 10bp deletion leading to a frame shift, and loss of a *KpnI* restriction site. PCR genotyping of targeted allele described above.

### Statistics

Statistical analysis was performed using Microsoft Excel (Microsoft) and GraphPad Prism software (GraphPad Software). Data sets were tested for differences using the unpaired *t* Test (for single comparisons), ANOVA followed by a Bonferroni’s post-hoc test or Tukey post-hoc tests (for multiple comparisons), Fisher’s exact (for proportions), Spearman *r* (for correlations), or Shapiro-Wilk (for normality). Non-normally distributed data sets were analyzed by Welch’s one way ANOVA. A *P* value of < 0.05 was deemed significant and in figures graded significance was designated as follows: *P* > 0.05 (ns = non-significant), *P* < 0.05 (*), *P* < 0.005 (**), *P* < 0.0005 (***), and *P* < 0.0001 (****). Error bars in graphs represent standard error of the mean (SEM).

## Supporting Information

S1 Fig *ret*^*hu2846*/+^ larvae exhibit phenotypic variation. 7dpf larvae immunostained with the HuC/D antibody to visualize ENS neurons in WT (A) and *ret*^*hu2846*/+^ (B-D) larvae. Asterisks indicate end of the gut tube (anal pore), and arrows denote the position of the most distal HuC/D^+^ neuron. At 7dpf, WT (*ret*^+/+^) larvae exhibit HuC/D^+^ ENS neurons along the full length of the gut (A). Some *ret*^*hu2846*/+^ larvae also show full colonization of the gut by HuC/D^+^ ENS neurons (B), and are referred to as ‘non-phenotypic’ or *ret*^*hu2846*/+^ NP. Other *ret*^*hu2846*/+^ larvae show varying extents of distal regions lacking HuC/D^+^ ENS neurons (C, D), and are referred to as ‘phenotypic’ or *ret*^*hu2846*/+^ P. High-resolution images of full gut length was constructed by tiling two overlapping micrographs.
(TIF)

S2 Fig Novel transgenic tools allow labeling of ENS progenitors and ENS neurons. SAGFF234A is a transgenic line with the Gal4 expressing gene trap construct integrated within the *chrm2a* gene on the chromosome 4 encoding the cholinergic receptor, muscarinic 2a. When crossed to transgenic fish carrying the GFP reporter gene downstream of the Gal4 recognition sequence (UAS:GFP) generates transgenic embryos expressing GFP within the ENS at 4dpf (A, B), in both ENS progenitors at 40hpf (C, arrows) and in virtually all HuC/D^+^ ENS neurons at 4dpf (D). (E) At 7dpf GFP can be detected in cellular processes of HuC/D^+^ ENS neurons, distal intestine shown (cellular processes of ENS neurons indicated with arrows). The high-resolution image of the full gut length (in D) was constructed by tiling two overlapping micrographs.
(TIF)

S3 Fig Proliferation, cell death and differentiation are all unaffected in *ret*^*hu2846*/+^ larvae. (A-C) Proliferation of ENS progenitor cells is equivalent in 48hpf WT (A) and *ret*^*hu2846*/+^ (B) larvae, shown using GFP from SAGFF234A;UAS:GFP to label pixel area of ENS progenitors and BrdU (following 30 minute BrdU pulse) to quantify proliferating cells per 220um (1000 pixels) of the distal migratory wavefront of progenitors (p = 0.9182) (C). The area (in pixels squared) (D) and roundness (4*area/(π*major axis^2^)) (E) of the migratory wavefront was quantified, and significant increases in both parameters reflect the fact that the *ret*^*hu2846*/+^ embryo wavefront is not elongated as is seen in WT (p<0.0001 for both area and roundness). (F-I) TUNEL staining on 42hpf WT (F,G) and *ret*^*hu2846*/+^ (H,I) larvae does not show any TUNEL^+^ apoptotic cells (red, G,I) within the GFP^+^ migratory stream of ENCCs (F,H). (J-M) Differentiation was assayed in the SAGFF234A;UAS:GFP line by comparing HuC/D expression with GFP expression in 4dpf larvae (J,K) and counting differentiated serotonergic cells (5-HT^+^) within 5dpf larvae (L,M). While HuC/D^+^ cells show expected significantly decreased numbers in *ret*^*hu2846*/+^ larvae (J, L, p = 0.0096 and p = 0.0045, respectively), the percentage of progenitor cells (GFP^+^Hu^-^) cells is not significantly altered (K, p = 0.3785), and the percentage of 5-HT^+^ cells is also not significantly altered (M, p = 0.2188).
(TIF)

S4 Fig *mapk10* is expressed within the SAGFF234A;UAS:GFP expressing ENS lineage. RNA *in situ* hybridization for *mapk10* was conducted in SAGFF234A;UAS:GFP embryos at 54 (A) and 72hpf (D). Immunostaining for GFP identifies the stream of migrating ENCCs (B, both streams shown, E, one stream shown). Merged images indicate that cells containing *map10* signal localize to the GFP+ population (C, F arrows denote a number of these cells).
(TIF)

S5 Fig Tools used for *mapk10* loss of function experiments. (A) Morpholino oligo (MO) gene knockdown of *mapk10*. Genomic organization of *mapk10* (*mapk10-001* from Zv9:CU651624.3:8583743:8720716:1) showing the position of the *mapk10* splice blocking MO (*mapk10* MO) at the intron3/4-exon4 boundary, which leads to excision of exon 4 (underlined). RT-PCR of injected embryos identifies loss of the 170bp exon4 and reduction of RT-PCR product from 500bp to 330bp, which was confirmed by sequencing. Exon 4 excision leads to a frame shift and early truncation of the protein at amino acid (AA) 25, leading to loss of the protein kinase catalytic domain and all Mapk10/Jnk conserved domains. (B) A novel *mapk10* mutant line has been generated by TALEN gene editing. TALEN targeting of exon 4 leads to the generation of a 10 base pair deletion in exon4, which leads to loss of a Kpn1 site, a frame shift, and early truncation of the protein at AA64.
(TIF)

S1 Movie Migration of the ENCC population in WT embryos from 48–56hpf. Depth coded Z-projected recording of GFP^+^ ENCC cells of *ret*^+/+^;SAGFF234A;UAS:GFP embryos from 48–56hpf with a frame interval 374 seconds.
(AVI)

S2 Movie Migration of the ENCC population in *ret*^*hu2846*/+^ embryos from 48–56hpf. Depth coded Z-projected recording of GFP^+^ ENCC cells of *ret*^*hu2846*/+^;SAGFF234A;UAS:GFP embryos from 48–56hpf with a frame interval 374 seconds.
(AVI)

S3 Movie Intestinal Motility in WT larvae. Recording (at 2.5 frames per second) of spontaneous intestinal motility of a WT larvae at 7dpf. The spatiotemporal map constructed from this original movie is shown in Fig 3. Movie is sped up 20 times.
(AVI)

S4 Movie Intestinal Motility in *ret*^*hu2846*/+^ larvae. Recording (at 2.5 frames per second) of spontaneous intestinal motility of a *ret*^*hu2846*/+^ larvae at 7dpf. The spatiotemporal map constructed from this original movie is shown in Fig 3. Movie is sped up 20 times.
(AVI)

S5 Movie Intestinal Motility in *ret*^*hu2846*/*hu2846*^ larvae. Recording (at 2.5 frames per second) of spontaneous intestinal motility of a *ret*^*hu2846*/*hu2846*^ larvae at 7dpf. The spatiotemporal map constructed from this original movie is shown in Fig 3. Movie is sped up 20 times.
(AVI)
