# Supplementary figures and images for: A Novel Zebrafish ret Heterozygous Model of Hirschsprung Disease Identifies a Functional Role for mapk10 as a Modifier of Enteric Nervous System Phenotype Severity
Source: PLoS Genet. 2016 Nov 30;12(11):e1006439. doi: 10.1371/journal.pgen.1006439 (PMC5130169; doi:10.1371/journal.pgen.1006439)

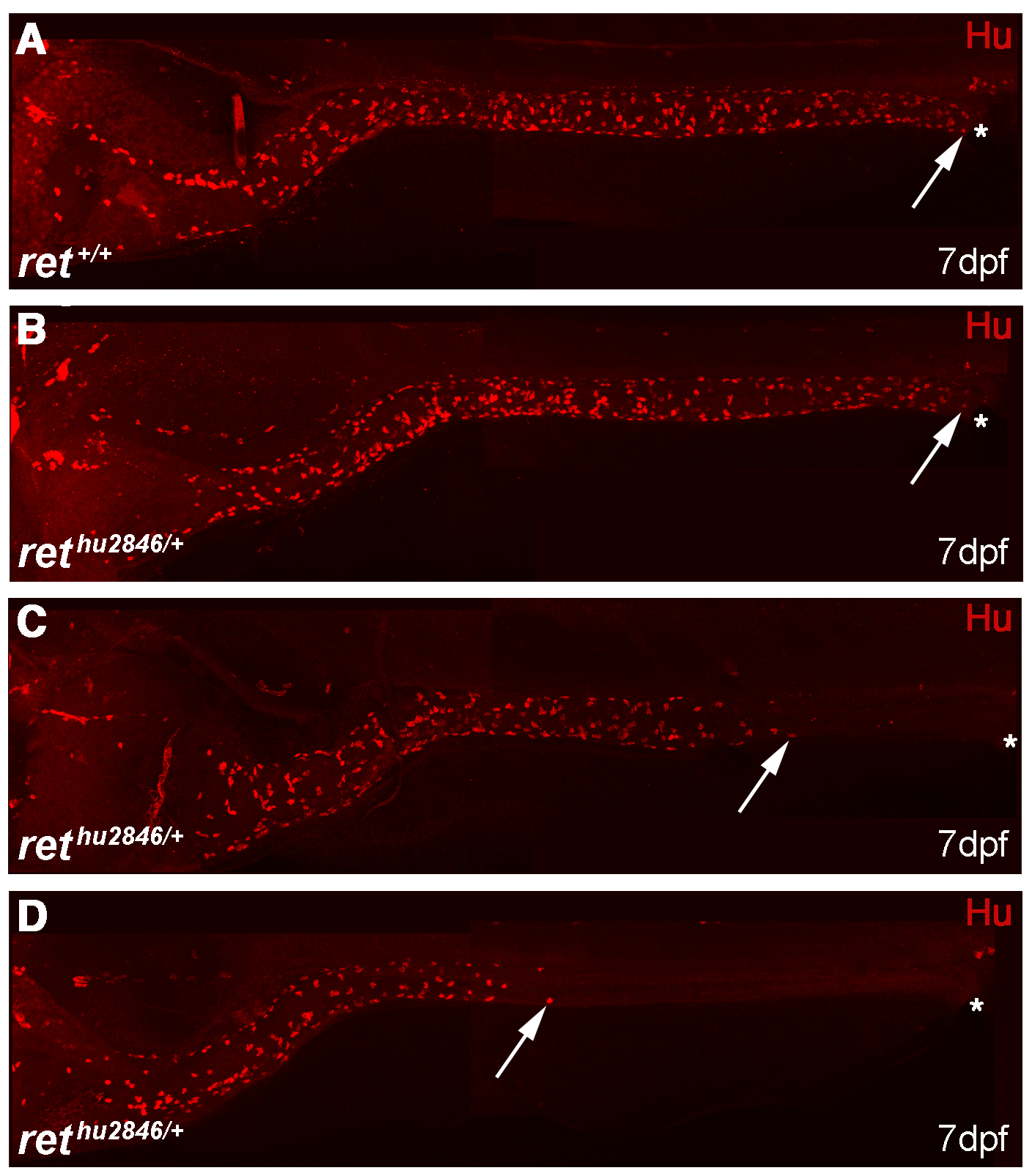

Supplement: S1 Fig — 7dpf larvae immunostained with the HuC/D antibody to visualize ENS neurons in WT (A) and rethu2846/+ (B-D) larvae. Asterisks indicate end of the gut tube (anal pore), and arrows denote the position of the most distal HuC/D+ neuron. At 7dpf, WT (ret+/+) larvae exhibit HuC/D+ ENS neurons along the full length of the gut (A). Some rethu2846/+ larvae also show full colonization of the gut by HuC/D+ ENS neurons (B), and are referred to as ‘non-phenotypic’ or rethu2846/+ NP. Other rethu2846/+ larvae show varying extents of distal regions lacking HuC/D+ ENS neurons (C, D), and are referred to as ‘phenotypic’ or rethu2846/+ P. High-resolution images of full gut length was constructed by tiling two overlapping micrographs. (TIF) [file pgen.1006439.s001.tif]

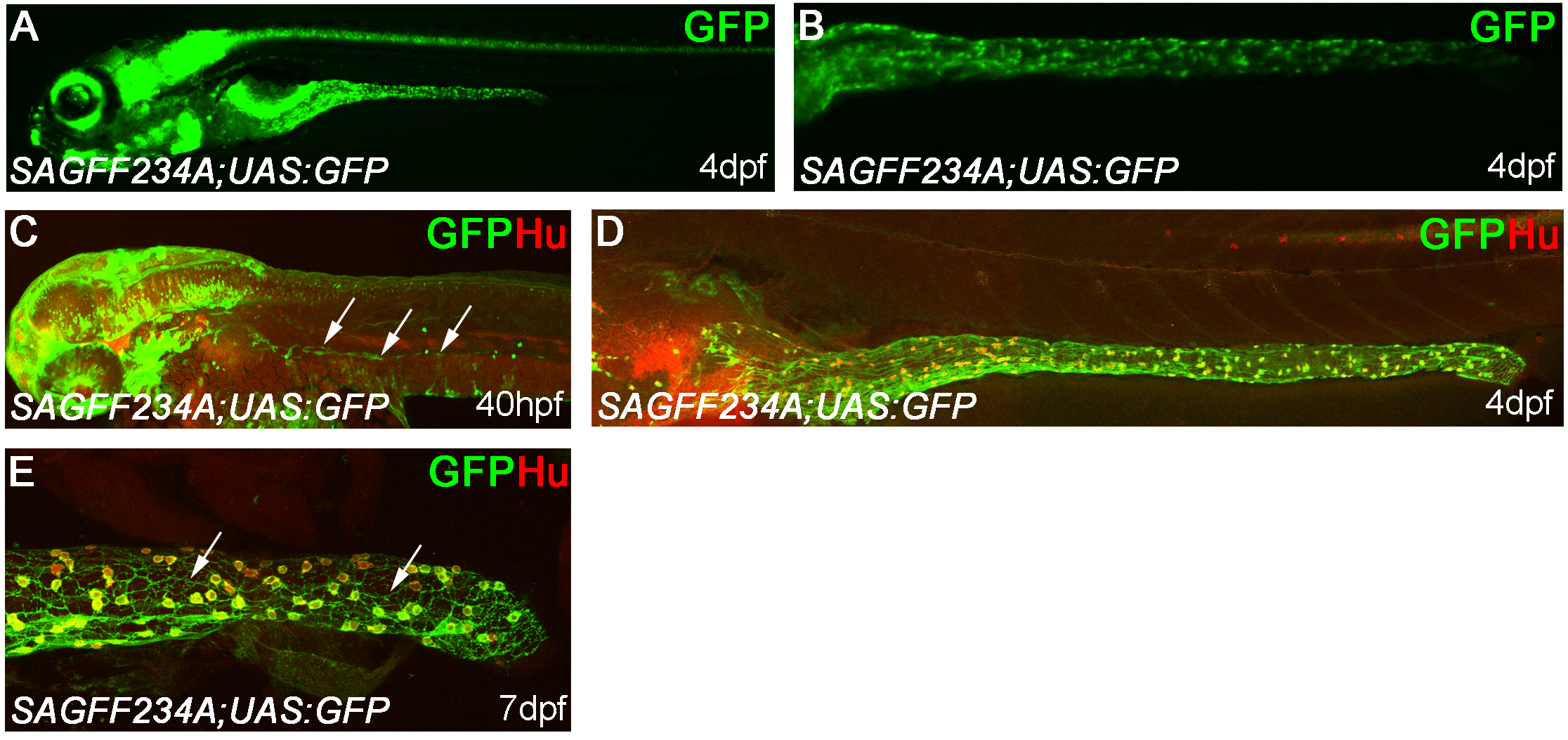

Supplement: S2 Fig — SAGFF234A is a transgenic line with the Gal4 expressing gene trap construct integrated within the chrm2a gene on the chromosome 4 encoding the cholinergic receptor, muscarinic 2a. When crossed to transgenic fish carrying the GFP reporter gene downstream of the Gal4 recognition sequence (UAS:GFP) generates transgenic embryos expressing GFP within the ENS at 4dpf (A, B), in both ENS progenitors at 40hpf (C, arrows) and in virtually all HuC/D+ ENS neurons at 4dpf (D). (E) At 7dpf GFP can be detected in cellular processes of HuC/D+ ENS neurons, distal intestine shown (cellular processes of ENS neurons indicated with arrows). The high-resolution image of the full gut length (in D) was constructed by tiling two overlapping micrographs. (TIF) [file pgen.1006439.s002.tif]

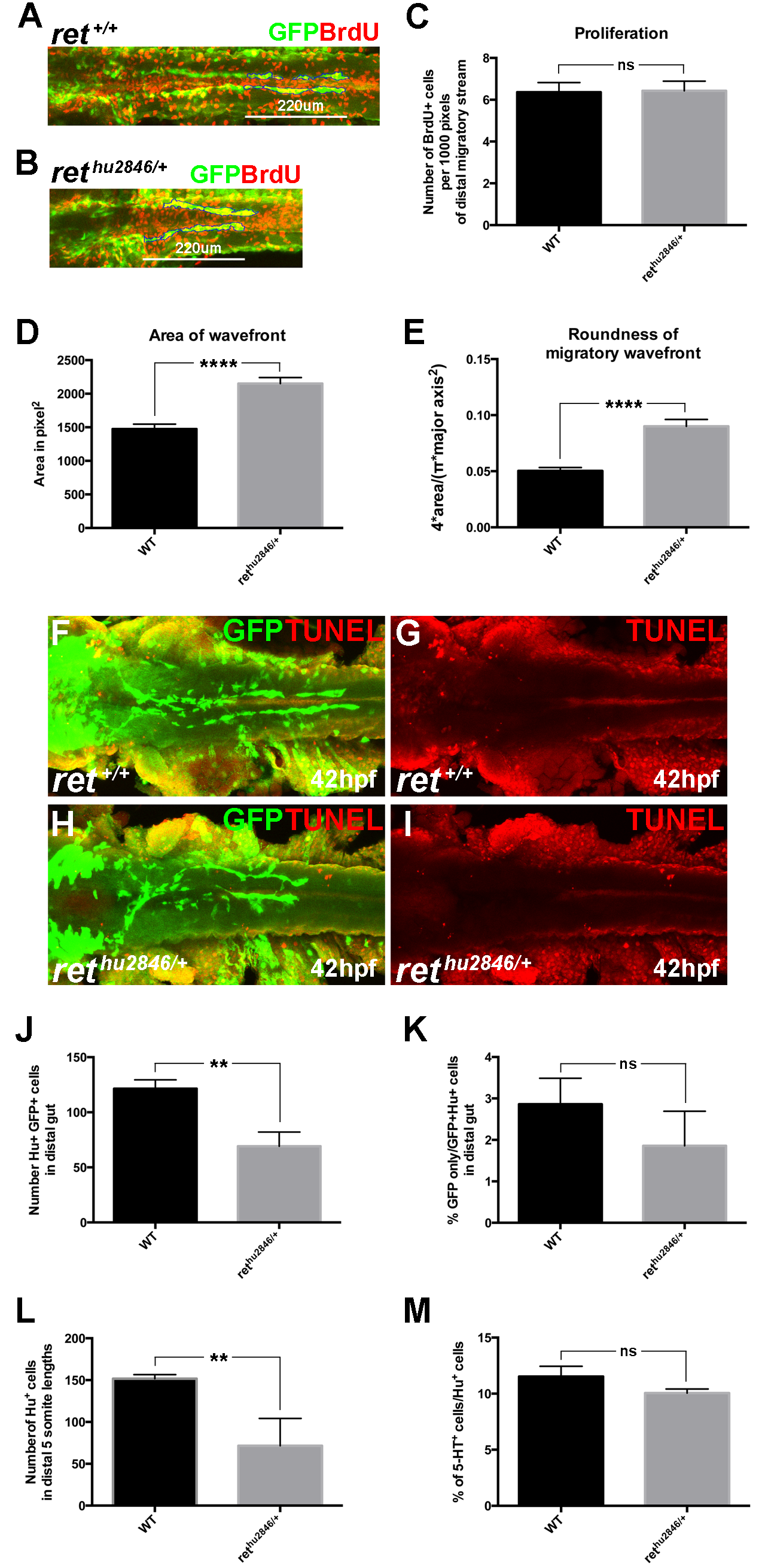

Supplement: S3 Fig — (A-C) Proliferation of ENS progenitor cells is equivalent in 48hpf WT (A) and rethu2846/+ (B) larvae, shown using GFP from SAGFF234A;UAS:GFP to label pixel area of ENS progenitors and BrdU (following 30 minute BrdU pulse) to quantify proliferating cells per 220um (1000 pixels) of the distal migratory wavefront of progenitors (p = 0.9182) (C). The area (in pixels squared) (D) and roundness (4*area/(π*major axis2)) (E) of the migratory wavefront was quantified, and significant increases in both parameters reflect the fact that the rethu2846/+ embryo wavefront is not elongated as is seen in WT (p<0.0001 for both area and roundness). (F-I) TUNEL staining on 42hpf WT (F,G) and rethu2846/+ (H,I) larvae does not show any TUNEL+ apoptotic cells (red, G,I) within the GFP+ migratory stream of ENCCs (F,H). (J-M) Differentiation was assayed in the SAGFF234A;UAS:GFP line by comparing HuC/D expression with GFP expression in 4dpf larvae (J,K) and counting differentiated serotonergic cells (5-HT+) within 5dpf larvae (L,M). While HuC/D+ cells show expected significantly decreased numbers in rethu2846/+ larvae (J, L, p = 0.0096 and p = 0.0045, respectively), the percentage of progenitor cells (GFP+Hu-) cells is not significantly altered (K, p = 0.3785), and the percentage of 5-HT+ cells is also not significantly altered (M, p = 0.2188). (TIF) [file pgen.1006439.s003.tif]

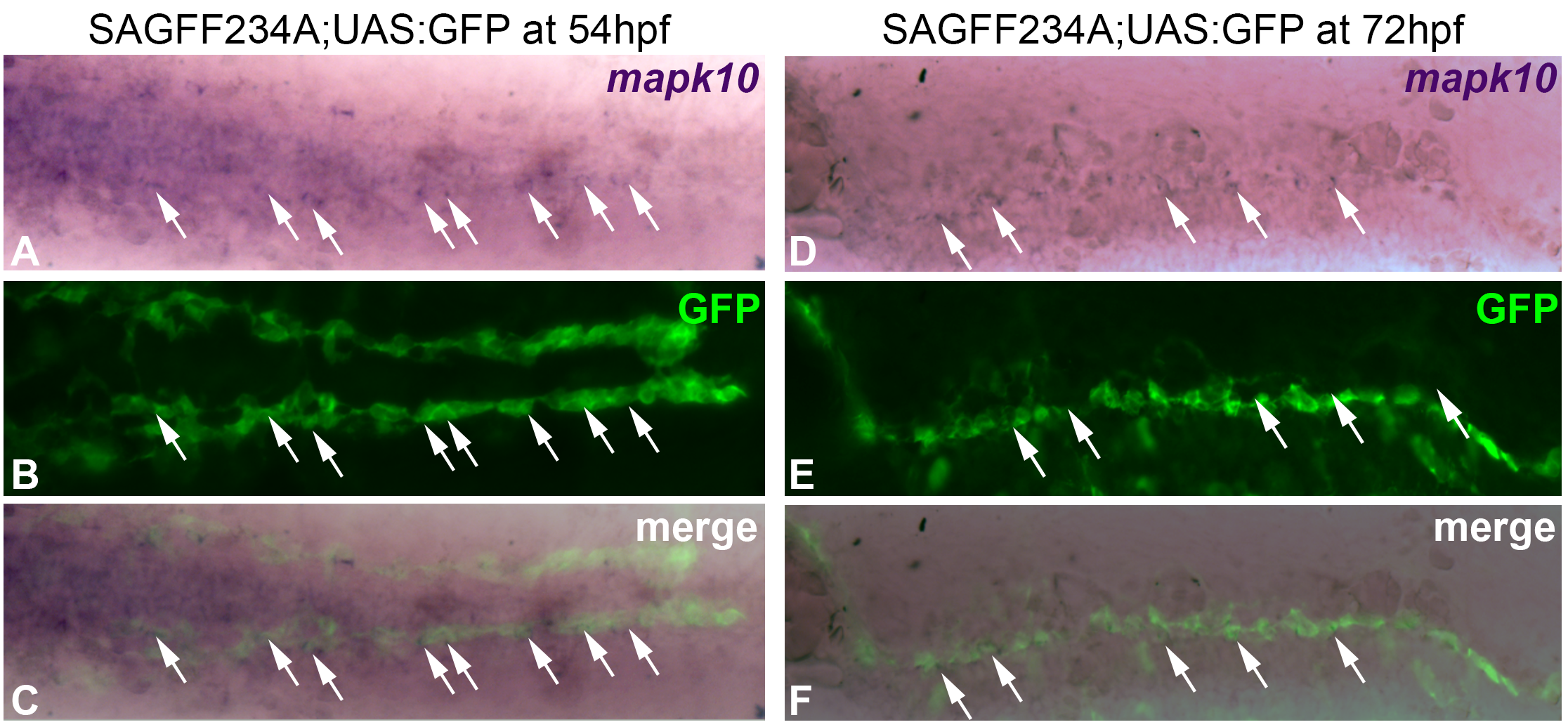

Supplement: S4 Fig — RNA in situ hybridization for mapk10 was conducted in SAGFF234A;UAS:GFP embryos at 54 (A) and 72hpf (D). Immunostaining for GFP identifies the stream of migrating ENCCs (B, both streams shown, E, one stream shown). Merged images indicate that cells containing map10 signal localize to the GFP+ population (C, F arrows denote a number of these cells). (TIF) [file pgen.1006439.s004.tif]

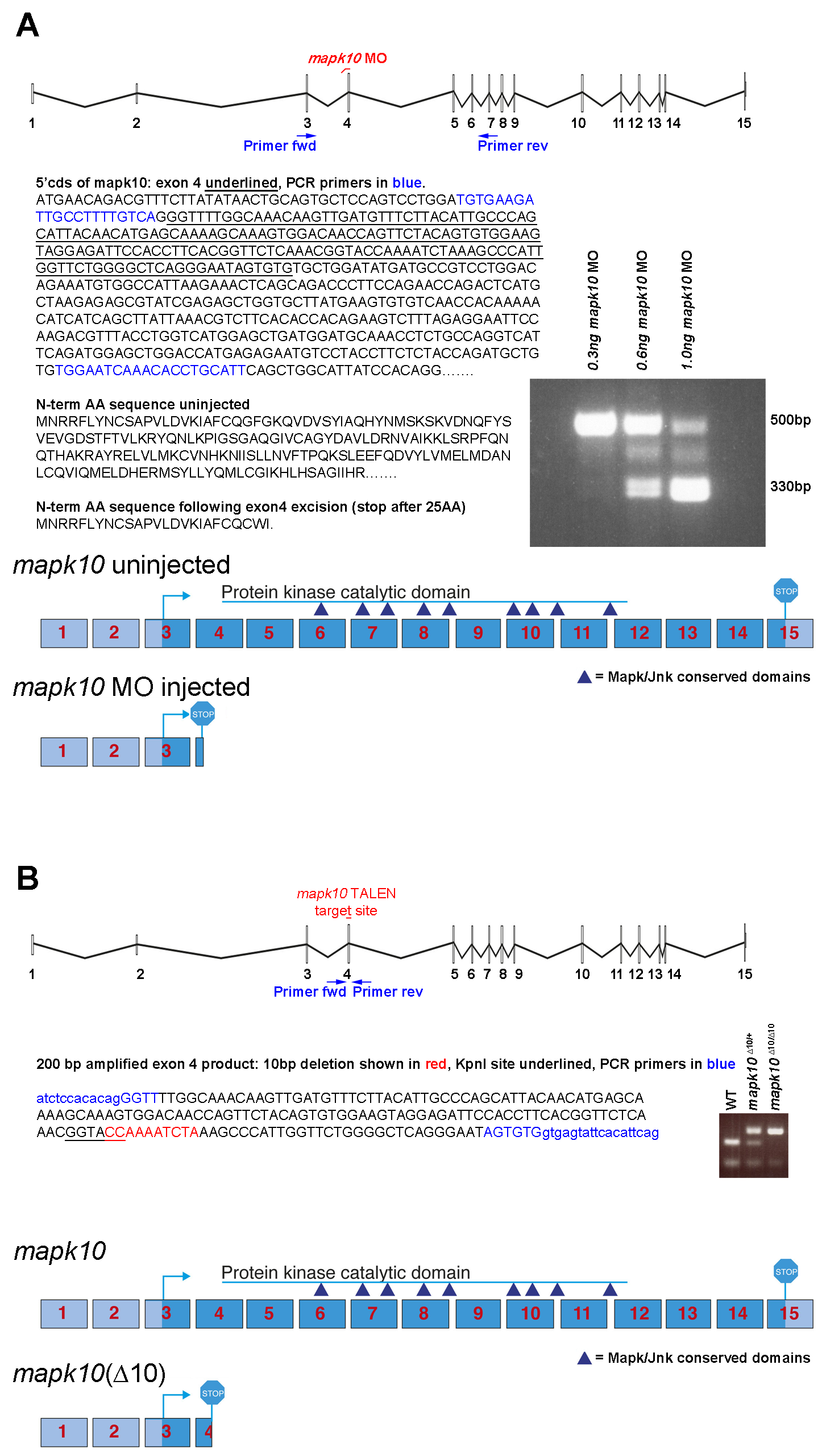

Supplement: S5 Fig — (A) Morpholino oligo (MO) gene knockdown of mapk10. Genomic organization of mapk10 (mapk10-001 from Zv9:CU651624.3:8583743:8720716:1) showing the position of the mapk10 splice blocking MO (mapk10 MO) at the intron3/4-exon4 boundary, which leads to excision of exon 4 (underlined). RT-PCR of injected embryos identifies loss of the 170bp exon4 and reduction of RT-PCR product from 500bp to 330bp, which was confirmed by sequencing. Exon 4 excision leads to a frame shift and early truncation of the protein at amino acid (AA) 25, leading to loss of the protein kinase catalytic domain and all Mapk10/Jnk conserved domains. (B) A novel mapk10 mutant line has been generated by TALEN gene editing. TALEN targeting of exon 4 leads to the generation of a 10 base pair deletion in exon4, which leads to loss of a Kpn1 site, a frame shift, and early truncation of the protein at AA64. (TIF) [file pgen.1006439.s005.tif]
